# Community detection with Greedy Modularity disassembly strategy

**DOI:** 10.1038/s41598-024-55190-7

**Published:** 2024-02-26

**Authors:** Heru Cahya Rustamaji, Wisnu Ananta Kusuma, Sri Nurdiati, Irmanida Batubara

**Affiliations:** 1 Department of Computer Science, Faculty of Mathematics and Natural Sciences, IPB University, Bogor, Indonesia; 2 Department of Mathematics, Faculty of Mathematics and Natural Sciences, IPB University, Bogor, Indonesia; 3 Department of Chemistry, Faculty of Mathematics and Natural Sciences, IPB University, Bogor, Indonesia; 4 Tropical Biopharmaca Research Center, IPB University, Bogor, Indonesia; 5 Department of Informatics, Faculty of Industrial Technology, UPN Veteran Yogyakarta, Yogyakarta, Indonesia

**Keywords:** Community detection, Greedy Modularity, Disassembly greedy, Computer science, Computational science

## Abstract

Community detection recognizes groups of densely connected nodes across networks, one of the fundamental procedures in network analysis. This research boosts the standard but locally optimized Greedy Modularity algorithm for community detection. We introduce innovative exploration techniques that include a variety of node and community disassembly strategies. These strategies include methods like non-triad creating, feeble, random as well as inadequate embeddedness for nodes, as well as low internal edge density, low triad participation ratio, weak, low conductance as well as random tactics for communities. We present a methodology that showcases the improvement in modularity across the wide variety of real-world and synthetic networks over the standard approaches. A detailed comparison against other well-known community detection algorithms further illustrates the better performance of our improved method. This study not only optimizes the process of community detection but also broadens the scope for a more nuanced and effective network analysis that may pave the way for more insights as to the dynamism and structures of its functioning by effectively addressing and overcoming the limitations that are inherently attached with the existing community detection algorithms.

## Introduction

A complex system consists of many interrelated elements and has complex and unpredictable relationships. In a network of complex systems, nodes represent elements and the edges reflect their interrelationships. Complex systems can be found in various fields, such as social, transportation, and biological systems. Network analysis is a valuable tool for understanding and analyzing complex systems. By representing the elements of a system as nodes and their relationships as edges, we can identify patterns and structures within the system that may not be immediately apparent from the raw data. This can help us to better understand the behavior of the system and possibly make predictions about its future behavior.

In network science, the community is one of the essential concepts in understanding complex systems because many real-world networks, such as social networks, biological networks, and transportation networks, exhibit community structures. A community can be defined as a group partition of the nodes in a network. A group of nodes in a partition is more likely to be connected than the nodes in other node groups^1^. Thus, the density of nodes in one group will be high. Communities in complex systems can be formed owing to the solid relationships between the components within the group. This relationship can take the form of an interaction, cooperation, or dependency. The elements in these communities often share common characteristics and objectives.

A community in a social network represents a group of friends who interact regularly. These people share the same hobbies or interests, such as a music community or a group of people living in the same neighborhood or city. In biological networks, such as protein-protein interactions or metabolic networks, communities can represent groups of proteins or metabolites that are functionally related or involved in the same biological process. In transportation networks, communities can represent groups of locations or nodes that are more likely to be connected than nodes outside the group based on geographic proximity, travel patterns, or land use.

The quality of the community formed by the community detection algorithm is a modularity^1^. If the modularity is high, it can be interpreted that community detection succeeded in grouping nodes into high-density communities that were functionally well-isolated. Conversely, suppose the resulting modularity is low. In this case, it can be interpreted that community detection was not successful in grouping nodes into high-density communities. The algorithm must determine how to partition the network in order to satisfy the desired community quality criteria. However, the network may have several partitions or non-optimal solutions.

Modularity, which is a key metric, was not the only metric considered. Predictability, which is based on link prediction, is a critical metric. It measures how well the community structure of a network can be derived based on the connectivity pattern in network^2^. In addition, the intuitive notion that distinct communities should be well-separated from the rest of the network is manifested in separability, which is another important measure of community quality^3^. Modularity was chosen in this study because it provides a clear and measurable way to assess the strength of a community structures. Modularity is widely accepted and applied in network analysis because it captures the density of links within and between communities. This strongly indicate the extent to which a network can be clearly separated into different groups or communities.

Determining the community structure of a network that maximizes modularity is an NP-hard problem. Determining an optimal solution for large networks within a reasonable time period is computationally challenging. The time required to determine the best modularity on a polynomial scale is immeasurable^4^. Determining an optimal solution is often not feasible, particularly for large-scale networks. Heuristic algorithms are often used to obtain a good or near-optimal solution within a reasonable amount of time. Heuristic algorithms use shortcuts or rules of thumb to find a solution that is close to the optimal solution without guaranteeing that it is the best possible solution. These algorithms are designed to be computationally efficient.

An essential problem in network analysis is the optimization of community detection algorithms. In particular, the Greedy Modularity algorithm applied to solve this problem tends to be stuck in local optima, which constrains its efficiency. This leads to a significant reduction in modularity, which is, an indicative primary metric that addresses with the strength of the community structure in networks. The aim of our study was to address this critical issue by exploring ways to enhance the Greedy Modularity algorithm to avoid such local optima and enhance the overall modularity in the detected community structures. We propose a novel refinement approach for the Greedy Modularity algorithm that integrates the latest exploration strategies such as disassembly node techniques and community strategies. More importantly, this methodological improvement not only avoids the limitations of conventional Greedy Modularity, but also greatly enhances its performance in detecting more accurate and meaningful community structures in complex networks. Our approach can overcome the usual limitations of the algorithm and thus provides a robust and efficient solution for community detection in many types of networks. This contribution is poised to have a significant impact within the field of network analysis, pointing towards a new direction for future research as well as its application.

## Related work

Previous studies have attempted to solve the community detection problem using different approaches. There are several approaches to community detection including mathematical models, networks, modularity, and evolutionary computing. Mathematical models are formal representations of a system that are described in terms of mathematical equations or algorithms, such as statistics^5^, matrix factorization^6^, and fuzzy^7^. The network approach is a strategy used to analyze and understand the structure and behavior of a network, such as local communities^8^, network embedding^9^, and cliques^10^. The modularity strategy optimizes community quality^11^, such as Louvain^12^, Leiden^13^, Girvan Newman^14^ , and Greedy modularity^15^. Evolutionary computational strategies are abstractions from the biological evolutionary theory used to create optimization procedures or methods. This strategy combines the concept of biological evolution with computer technology such as genetic algorithm^16^ and particle swarm optimization^17^.

However, many algorithms e used to achieve this maximum modularity provide suboptimal solutions. In addition, some algorithms generate small or large communities that may not be relevant to the actual context. Some algorithms, such as adding or removing nodes or edges, are less robust to network changes. These strategies produce different results when they are applied to a network for community detection. Each algorithm provides different community results and different modularity^18^.

One of the well-known algorithms and many references for solving community detection problems is the Greedy Modularity algorithm proposed by Newman^15^. This algorithm is a heuristic that searches for an optimal modularity value at each exploitation stage. This algorithm forms a community by combining two previously created communities to increase modularity. The iteration stops when there are no more communities to join to improve modularity. However, this algorithm can be trapped in a local optimum that is far from the global optimum. The Greedy Modularity algorithm focuses only on the best solution at each step, and is not the best solution for the entire problem. The algorithm may not be able to find communities with higher modularity than the solution it finds. Therefore, the modularity generated by the Greedy Modularity algorithm may not always be high, depending on the network conditions.

Several factors can lead to local optima for community detection. Modularity-based community detection methods may suffer from a resolution limit, which means that they may fail to detect small communities (e.g., cliques and triads) within larger communities^19^. Consequently, modularity-based methods tend to merge small communities into larger ones, resulting in loss of information about the network structure. Researchers have proposed various approaches to address the resolution limit problem, such as the use of generalized modularity density and z-score-based modularity. The generalized modularity density is a method that can detect communities of different sizes and shapes by considering the density of nodes in the network^20^. Z-score based modularity is another approach that can detect communities of different sizes by normalizing the modularity score^21^.

Another influential factor is weak community structures^22^. Researchers have proposed various approaches to address this issue, such as hidden community detection and weak supervision. Hidden communities refer to weak or disguised communities that are not easily detected using traditional community detection methods^23^. Weak supervision of community structures is another approach that can detect communities of different sizes and shapes by using the node2vec algorithm^24^. Communities with low embedding also contribute to difficulties in identifying communities^25^. This problem can be addressed using network embedding methods, which aim to project nodes of the same community close to each other in a geometric space where they can be detected by standard data clustering algorithms^26^. However, it is essential to choose appropriate parameters for embedding techniques to achieve optimal performance.

## Materials and methods

### Dataset

In this study, real-world and synthetic datasets were used for the community detection. Real-world datasets were used: Zachary Karate Club, Dolphin, Les Miserable, Polbooks, Adjnoun, Football, information retrieval, hardware architecture, and a database. Meanwhile, we used the synthetic Lancichinetti–Fortunato–Radicchi (LFR) dataset, whose characteristics mimic real-world networks, such as community structure and degree distribution^27^.

### Methods

#### The Greedy Modularity algorithm

Consider an undirected graph $G = (V, E)$ with a set of vertices *V* and a set of edges *E*. Let *n* be the number of vertices in *G*, and *m* be the number of edges in *G*. Consider a graph *G* and let *A* be its adjacency matrix. Specifically, $A_{ij}$ represents the number of edges connecting nodes *i* and *j*. If nodes *i* and *j* are not connected, then $A_{ij}$ is equal to 0, denoted as $(i, j) \notin E$.

The Greedy Modularity algorithm^15^ on *G* starts by creating *n* communities where each community consists of one node. In this case, let $C_i$ be the community of node $i \in V$. The second step combines the two communities with the highest increase in modularity using Formula 1 where $e_ij$ is the edge in the network connecting a node in community *i* to a node in community *j*, and $a_i$ denotes the number of edges in community *i*. This step is performed until there are no more network partitions with a higher modularity.

1 $$\begin{aligned} \Delta Q = 2\left( e_{ij}-a_i a_j\right) \end{aligned}$$

Modularity was used to measure the community quality. The network modularity *Q*(*S*) is calculated as the sum of the modularity of each community, as expressed by Formula 2, where $l_c$ and $k_c$ denote the number of edges and vertex degrees in community $c \in S$, respectively. At the same time, L is the number of edges and vertices in the network as a whole.

Modularity was the primary strategic and central metric of our study. Modularity does not, on any account, stand as a measure of the density of links within communities versus each other versus between them. Instead, it stands as an underlying indicator of the structure and efficacy of a network. The significance of our research lies in its ability to reveal the nuances of community interaction and segregation, which is crucial for understanding and amplifying the underlying patterns and behaviors of these complex networks. We attempt to reflect on how our modified Greedy Modularity algorithm boosts modularity to detect efficient complex community structures. This is essential because high modularity reflects good communities and more precisely divided communities, leading to better real-world application and general views. Therefore, it is more than a metric but rather a perspective from which the quality and relevance of our algorithm outcomes are examined and evaluated. In this study, modularity was used to evaluate the superior performance of our improved Greedy Modularity algorithm over the current models, illustrating its enhanced ability to identify more coherent and meaningful community structures. This decision was motivated by our commitment to offer a complete and scientifically rigorous approach for detecting communities in complex networks.

2 $$\begin{aligned} Q\left( S\right) = \sum _{c \in S} \left( \frac{l_c}{L}-\left( \frac{k_c}{2L}\right) ^2 \right) \end{aligned}$$

**Figure 1:**
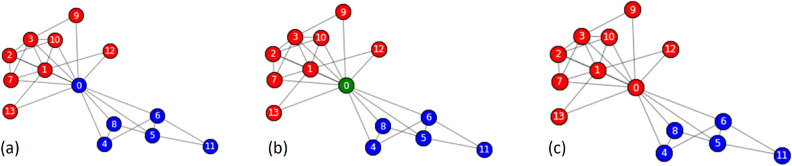
Node exploration, node disassembly (**a**) Two communities are formed, blue and red (**b**) Node 0 is removed from the blue community (**c**) Node 0 joins the red community.

Greedy Modularity was explored to achieve the highest modularity value in each iteration. However, it can be trapped at the local optimum therefore we required a correction step in the form of exploration. Exploration involves looking for alternatives to avoid local optima. The basic concept of this algorithm is to obtain a balance between exploration and exploitation^28^. The exploration carried out is release nodes, namely disassembly nodes in a community and disassembly community. Figure 1 illustrates node exploration. The community formed by the Greedy Modularity algorithm consists of two communities, blue and red. It can be seen that node 0 is a node with low embeddedness and is a weak node. Therefore, the node is removed from the blue community such that node 0 forms a new green community. In the next iteration, node 0 joins the red community, producing a community with better modularity.

**Figure 2:**
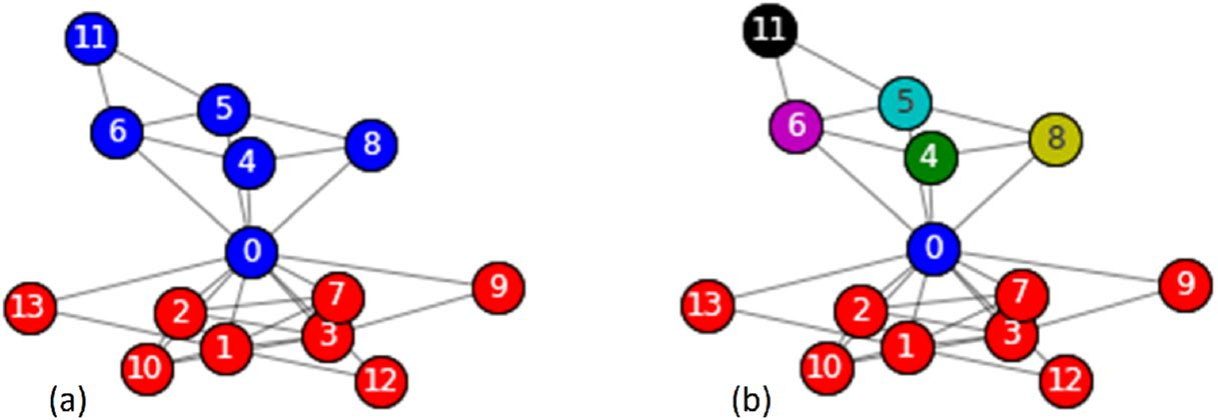
Community exploration, community disassembly (**a**) two communities are formed, blue and red (**b**) the blue community is disassembled, and each node becomes a community.

Community exploration was performed by disassembling the communities (Fig. 2). The blue community is weak because it has a weak node, that is node number 0. The community is disassembled such that each node in the blue community becomes a community, which is exploited in the next iteration.

The Greedy Modularity algorithm comprises three steps. The first part is the community initialization. In other words, each node is made into a community. If there are *n* nodes, then *n* communities are formed. The second part is the exploitation stage, which combines nodes that can potentially increase modularity iteratively. This phase was a greedy algorithm. The third part is the exploration stage, which comprises of two alternatives. The first is disassembling the nodes in a selected community to become a community. The second alternative is a disassembly community, that disassembles a community and each node into a community. After disassembling the node, it returns to the second stage, recombining the split nodes and communities to increase the modularity. The possibility of exploration and exploitation in each iteration was expressed as a percentage.

We have developed various algorithms for node and community disassembly. The purpose of these strategies is to improve Greedy Modularity. The datasets used are real-world standard benchmark datasets that are publicly accessible and generate synthetic data that are often used in research on community detection research.

#### Node disassembly strategy

The node disassembly strategy is implemented such that nodes in the network that have the potential to move communities are disassembled from the community to which they belong. There are four strategies for disassembly nodes: the disassembly of random nodes, weak nodes, nodes with low embeddedness, and disassembly nodes that do not form triads.

The strategy of disassembling random nodes is performed by randomly selecting nodes with two or more nodes in one of the communities $(|C| > 1)$. The selected node $v^*$ is then transformed into a single community. Finally, each connected component of the rest iss transformed into a community, as described in Algorithm 1.

The main steps of time consumption in Algorithm 1 are the random selection of a community and formation of new communities from the connected components. The corresponding time complexity for the initial selection of a worst-case community $C^*$ from graph *G* based on the condition $|C|>1$ is *O*(*n*), where each node forms its community, presupposing n total nodes. The subsequent operations are *O*(1) processes composed of a random selection of the $v^*$ node from $C^*$ and creation of a single-node community. However, the critical step of forming new communities from the remaining connected components after removing $v^*$ from $C^*$ yields the worst-case complexity of *O*(*k*), where k denotes the number of nodes in $C^*$. With $k \ll n$, the algorithm can be estimated as *O*(*n*). The algorithmic steps are simple and require basic code implementation. Its primary operation is to select a random community and then a node within it, which is computationally inexpensive (*O*(1)).

**Algorithm 1:**
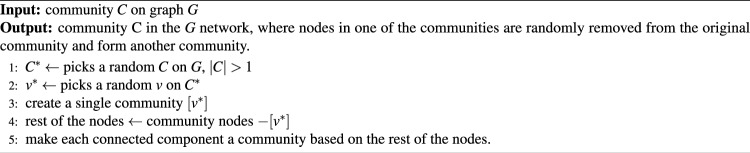
The algorithm disassembly random nodes.

The strategy for disassembling weak nodes in a weak community is given by Eq. (3). In a strong community, each node has an internal degree greater than the external degree. If there is a node in a community that has an internal degree $k_i^{int}(C)$ less than or equal to the external degree $k_i^{ext}(C)$, the community is weak^1^. Automatically, a weak node weakens a community. The first step of the algorithm is to compute the internal and external degrees of each node $v \in V$ . The next step is to determine which node $v^*$ in the community has an external degree greater than the internal degree. For the selected node to be in the formed community, we added the condition that the internal degree of the node is $\ge 2$. The chosen node $v^*$ is then transformed into a single community. Finally, each connected element of the rest is transformed into a community. The procedure is described in Algorithm 2.

The algorithm for disassembling the weak nodes in a weak community displays an interesting complexity profile. One part of the algorithm that incurs its most significant computational burden *O*(*nd*), involves iterating over every node (*n*) and examining each of its incident edges out of a maximum of any node in the graph (*d*). Thus, a detailed examination should be used to exactly identify the internal and external degrees of every node—an especially vital step in identifying weak nodes—those whose internal connectivity within their community is outweighed by their external connections. A significant strength of this algorithm is that it explicitly attacks and dismantles weak nodes among communities. Considering only those nodes of the network whose internal degree is smaller than or equal to that of its external, one effectively finds sections of the network that may disrupt the connectivity or overall integrity of the community structure. It is most applicable in networks where the failure of weak links or nodes significantly undermines the network’s robustness and functionality.

3 $$\begin{aligned} i \in C,k_i^int \left( C\right) \le k_i^ext \left( C\right) \end{aligned}$$

**Algorithm 2:**
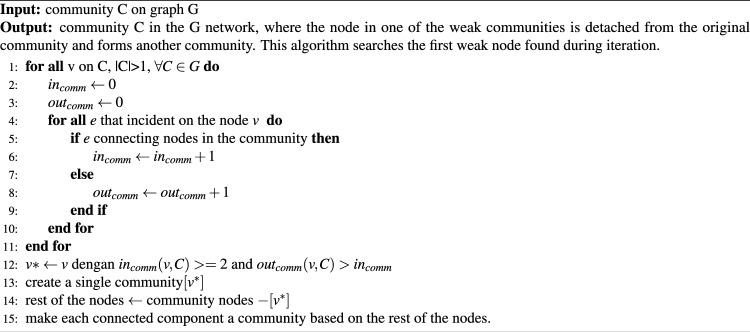
Weak node disassembly algorithm on weak community.

The strategy for disassembling nodes with low embeddedness values is expressed in Eq. (4). Embeddedness measures the integration of nodes into a network community. The lower a node’s embeddedness in a community, the fewer links or connections it has with other nodes. Nodes with a lower embeddedness can be used to identify more vulnerable communities. The lower the embeddedness of a node in the community, the lower the degree $k_v$ ratio of neighboring nodes in one community compared to all neighbors^29^. The first step of the algorithm is to compute the embeddedness of each node $v \in V$. The next step is to form a single community at node $v*$, that has the lowest embeddedness. Each connected component of the remaining nodes is converted into a community, as described in Algorithm 3.

Disassembly nodes with low embeddedness algorithms incur significantly in the embeddedness calculation of every node by checking the degree to which nodes exhibit in their communities against the number of interactions that exist for them. Finally, this calculation is performed for each node present in a community, and it shows some complexity of *O*(*nk*) where n is the number of nodes and k is the average number of neighbors per node. The other steps involve identifying a node with minimum embeddedness and reassignment into a new community. The utility of the algorithm lies in removing nodes with low embeddedness from their current communities and placing them in new communities, where they provide a better contribution of behavior more efficiently for modularity. Such strategic reassignment is expected to enhance the overall cohesion and modularity information of the network, establishing a systematic way to optimize community structures and improve upon the representational accuracy of the network, particularly the context within which the network integration of communities as well as clear delineation is essential.

4 $$\begin{aligned} f(v,C)= \frac{k_v^C}{k_v} \end{aligned}$$

**Algorithm 3:**
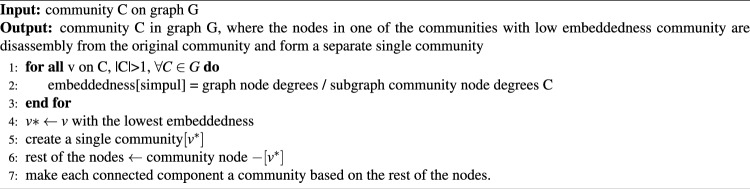
The algorithm disassembly nodes with low embeddedness.

Disassembly nodes that do not form a triad. A triad consists of three nodes that are connected to an edge in a community or network, as expressed in Eq. (5). Triads can show strong associations between nodes in a community and are used to identify communities by identifying the subgraphs that constitute a triad. The first step of this algorithm is to form a subgraph for each community with $|C|>4$ and select nodes that do not constitute a triad. The next step is to create a single community at node $v^*$, which is a node that does not form a triad. Each connected component of the remaining nodes was converted into a community as specified in Algorithm 4.

This algorithm was applied to strengthen the community structure within the networks by removing non-participating triad forming nodes. According to this definition, triads are a set of three interrelated nodes and are very useful in indicating strong intra-community relationships. This algorithm pass through every node in the community carefully and executes a combinatorial analysis that considers all possible triadic combinations. In a community of n nodes, this gives an $O(n^3)$ level of computational complexity, considering that multiple checks have been exhaustively placed on triad formation. This deeper scrutiny is critical for correctly identifying nodes with weaker community connections, which in turn allows the community structure to reflect more accurately, meaningfully and strongly expressed relationships between nodes. It is only this algorithm that is most useful in complex network analyses where the strength and clarity of community connections are necessary.

5 $$\begin{aligned} Triad=\bigl \{u:u \in S, \bigl \{\left( v,w\right) :v,w \in S,\left( u,v\right) \in E,\left( u,w\right) \in E,\left( v,w\right) \in E\bigr \} \ne \varnothing \bigr \} \end{aligned}$$

**Algorithm 4:**
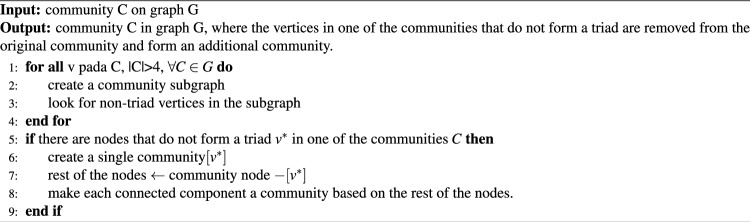
The algorithm disassembly nodes that don’t form a triad.

#### The community disassembly strategy

Five community disassembly strategies were used: random communities, weak communities, communities with low internal side densities, communities with low triad participation ratios, and communities with low conductance. The random strategy selects one of the communities formed in the network that has two or more nodes $(|C| > 1)$, and performs disassembly so that each node in that community becomes a separate community. This method is described in Algorithm 5.

As described in Algorithm 5, the random community disassembly algorithm is simple yet effective for reorganizing communities in various networks. This algorithm starts by randomly choosing a community $C^*$ from graph G, with the restriction that $C^*$ has more than a single node $(|C|>1)$. It then proceeds to iterate each node *v* in $C^*$, to reassign every node such that they have their communities. The computational complexity of this algorithm is *O*(*n*), which is linear to the number of nodes in the selected community $C*$. This linear complexity arises from the single-pass iteration over each of the nodes in $C*$, free of any nested or complex operations, thereby making the algorithm highly efficient, particularly for communities with fewer node counts. The primary benefit of the present algorithmic approach is its simplicity and rapid execution, such that it is the foremost contender during the initial analyses. It also enables the disbanding of a community in individual nodes such that each node is re-evaluated for community affiliation and boosts the modularity of a network. This aspect finds better use when the initial community assignments are either suboptimal or uncertain.

**Algorithm 5:**
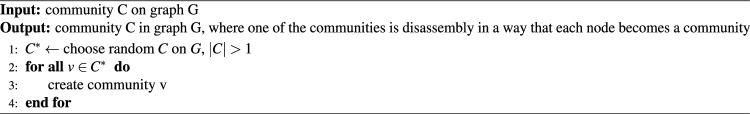
Random community disassembly algorithm.

A weak community is interpreted as one that is unstable and easily divided. In a social network, a weak community may lack social cohesion, a shared sense of purpose or values, or effective communication and collaboration among its members. The weak community is stated in 6 by relaxing the presence of nodes $\ i \in C,k_i^{int} \left( C\right) \le k_i^{ext} \left( C\right)$. The strategy of disassembling a weak community starts by creating a subgraph based on each community in *G*, and then computing the inside edge of each community and calculating the outside edge of the subgraph with respect to *G*. Choose the community with the smallest difference between the inner and outer edges. Disassembly was then performed such that each node in the community became a separate community. This strategy is described in Algorithm 6.

This algorithm contemplates the restructuring of networks through the disbanding of so-called weak communities, featuring a more significant number of external edges than internal ones. Mostly from the edge computations performed within every community, the complexity of the algorithm is $O(m + n)$ , where m and n are the numbers of edges and nodes, respectively. It is increased by the linear complexity from the selection of the weakest community, as well as the disassembly of its nodes, given an overall complexity that is beyond the linear but far below the quadratic for most network structures. The disassembly of these communities causes each node to join more cohesive communities iteratively, thereby enhancing the modularity of the network in general. This minimalist approach not only smoothes the network structure but also allows a strategic improvement in modularity by means of reallocation of the nodes to appropriate communities.

6 $$\begin{aligned} k_i^{int} \left( C\right) > \sum _{\left( i \in C \right) } k_i^{ext} \left( C\right) \end{aligned}$$

**Algorithm 6:**
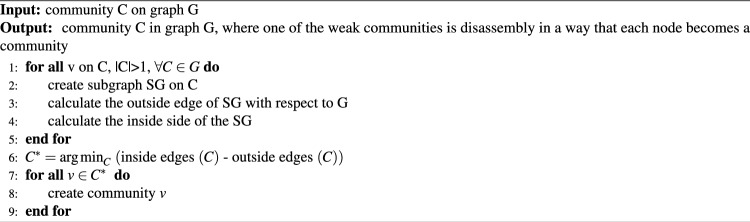
Weak community disassembly algorithm.

Select a community with a low internal edge density (Eq. 7), where $m_s$ is the number of edges in the community and $n_s$ is the number of vertices in the community. Communities with a low internal edge density in the network have few relationships or edges. In this case, the internal edge density indicates the closeness of the relationships between nodes in a community. Communities with low internal density indicate that the relationships between nodes in the community are less stable. Communities with high internal edge density are more stable because they have more connections between nodes, making it more difficult for them to be affected by network changes. The first step of this method is to create a subgraph based on each community in *G* and then calculate the density of the subgraph. Communities with the lowest density were selected for this study. Decomposition wass then performed so that each node in the community became a separate community. This strategy is described in Algorithm 7.

This algorithm strategically disassembles communities within a network that exhibits a low internal edge density. This measure is pivotal in evaluating community stability because it reflects the intensity of inter-node connections within itself. It begins by creating a subgraph for each community and calculating its density, an operation whose complexity depends essentially on the number of nodes and edges in each community, usually equal to $O(m + n)$. The next step involved identifying the community with the lowest density. Linear complexity was identified based on the total number of communities. Subsequently, the selected community is disbanded after being transformed into an independent community for every node. The advantage of this algorithm is that it helps increase modularity in networks. The nodes break away from weaker communities. Therefore, nodes can affiliate or form more integrated communities, thereby increasing the potential overall modularity of the network. This approach strengthens the network not only by resolving the weaker parts but also by aiding in reorganizing communities systematically, which is especially important for networks where the toughness and integration of communities pose contributing factors.

7 $$\begin{aligned} f\left( S\right) =\frac{m_s}{\left( \frac{n_s \left( n_s-1\right) }{2} \right) } \end{aligned}$$

**Algorithm 7:**
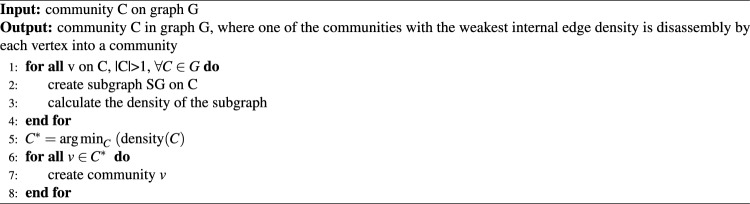
The algorithm disassembly the community with low internal edge density.

Choose a community with a low triad participation ratio (Eq. 8) where *u*, *v*, and *w* are vertices; *E* is an edge; and $n_S$ denotes the number of vertices in the community. The Triad Participation Ratio (TPR) is a metric used to measure the participation of nodes in triads in a social network. The TPR measures how often a node is involved in a triad, which is the basic unit in a social network consisting of three nodes and three relationships. In the community context, TPR determines how nodes in a community are involved in triads and affects the stability of the community. Nodes with a high TPR tend to have many connections with other nodes in the community and play an active role in the formation of triads. This community makes it more stable and less affected by changes in the network. Conversely, nodes with a low TPR tend to have fewer connections with other nodes in the community and play a less active role in the triad formation. This makes communities more vulnerable to changes in the network and is less stable. The lower the triad participation ratio, the lower the density, cohesiveness, and grouping^30^. The strategy is to create a subgraph based on each community in *G*, then count the number of nodes that form a triangle, and divide it by the number of nodes in that community to produce a triangle participation ratio score. The community with the smallest score was selected. Next, disassembly is performed such that each node in the community becomes a separate community, as shown in Algorithm 8.

The computational process of this algorithm implementation begins with the construction of subgraphs to enumerate the nodes that participate in the triads for every community. This enumeration is particularly complex given the density and efficiency of the triangle counting algorithm, which at most can be computationally heavy but always, in general, not above $O(n^3)$ for sparse networks. Most importantly, the algorithm leverages the concept of triad participation to improve and reinforce the community structure in a network, signifying that tightly and highly linked sets of nodes are important properties of an efficient and viable network topological structure.

8 $$\begin{aligned} f\left( S\right) = \frac{|\bigl \{u:u \in S,\bigl \{\left( v,w\right) :v,w \in S,\left( u,v\right) \in E,\left( u,w\right) \in E,\left( v,w\right) \in E\bigr \}\ne \varnothing \bigr \}|}{n_S} \end{aligned}$$

**Algorithm 8:**
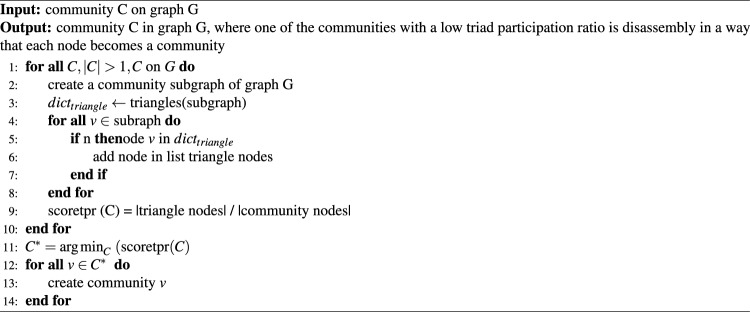
The algorithm disassembly the community with a low triad participation ratio.

Choose a community with high conductance as given in Eq. (9) where $m_{out}$ is the number of edges leaving the community and $m_c$ is the number of edges inside the community. Conductance measures how well a community is isolated from nodes and connections outside the community. Communities with high conductance have many nodes and relationships connected to the outside, resulting in a more open and heterogeneous community. A community has more diverse characteristics and goals and weaker connections between nodes within the community. Consequently, communities with high conductance tend to be more unstable and perform worse in terms of maintaining engagement and cohesion. The lower the conductance, the more well-established a community is, and it serves nearly perfectly in ordering communities from the most separable to the least separable^30^. First, a subgraph is created based on each community in *G*, then the inside and outside edges in the community are calculted, and the conductance score is computed based on Eq. (9). The community with the highest conductance score was selected for this study. Next, disassembly was performed such that each node in the community became a separate community as specified in Algorithm 9.

This opens up network modularity through the disassembly of communities with high conductance. The primary basis for the computational complexity of this algorithm is the creation of subgraphs based on every community and, subsequently, the computation of conductance scores. The complexity of forming subgraphs and tracing the internal and external edges in each community scale is $O(m + n)$, where m represents the number of edges. By contrast, n represents the number of nodes. This process is iterated over C communities and hence has an overall complexity of $O(C * (m + n))$. The calculation of conductance, which is the ratio of external to total edges, has a linear complexity with regard to the line count. Upon identifying the community with the largest conductance, this algorithm deconstructs this community by changing each node into a singleton community that will require extra complexity *O*(*n*). The strategic manner in which the algorithm handles communities with high conductance enhances the modularity of a network. In this manner, the algorithm effectively rearranges the network targeting communities with many external connections and weak internal connections to strengthen intra-community ties and delineate boundaries more clearly. Such reorganization increases the overall network cohesion and may potentially enhance the modularity score. Thus, it represents a robust and stable network architecture. This is a critical algorithm that refines the modular architecture of a network to emphasize the network stability and internal community strength.

9 $$\begin{aligned} f\left( S\right) = \frac{m_{out}}{2*m_c+m_{out} } \end{aligned}$$

**Algorithm 9:**
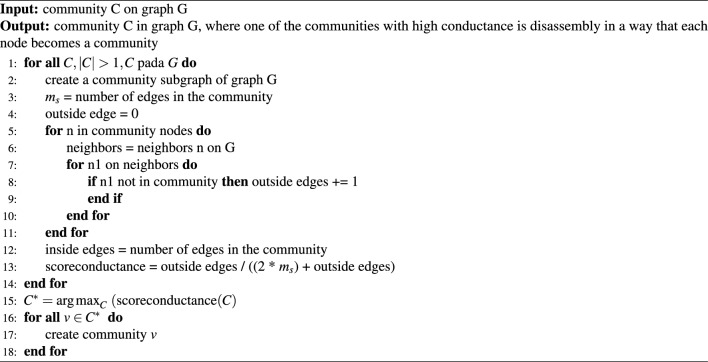
The algorithm disassemble the community with high conductance.

### Evaluation

Modularity evaluation is used to evaluate the quality of community detection in a network and how well a community detection algorithm can separate the network into several homogeneous parts. In addition, modularity can be used to compare the community detection results of different algorithms to select the most appropriate algorithm.

Normalized mutual information (NMI) is used to determine the amount of information related to two variables, which is normalized to a value between 0 and 1. The NMI is expressed in Eq. (10) where $P_{UV}(i,j)$ represents the probability of the value pair of communities *U* and *V*, and $log\frac{P_{UV}\left( i,j\right) }{P_U(i)P_V(j)}$ represents the logarithm of the ratio between the joint probability of communities U and V and the product of the probabilities of each community. Logarithmic values were used to measure the amount of information shared by the two variables. NMI is a normalized version of the mutual information obtained by dividing it by both the community entropy $-\sum _{i=1}^R P_U(i)log P_U(i)$ and $-\sum _{i=1}^R P_V(i)log P_V(i)$, which is used to facilitate comparisons between two different groupings. This can be used to compare the correlation between two distributions with different numbers of elements. Values close to 1 indicate a strong correlation between two variables, whereas values close to 0 indicate a weak correlation.

10 $$\begin{aligned} NMI(U,V)=\frac{2\sum _{i=1}^R\sum _{j=1}^C P_{UV}(i,j)log\frac{P_{UV}\left( i,j\right) }{P_U(i)P_V(j)}}{-\sum _{i=1}^R P_U(i)log P_U(i)-\sum _{i=1}^R P_V(i)log P_V(i)} \end{aligned}$$

For the external evaluation, the comparison algorithms were AGDL^31^, Fluid^32^, Belief^33^, CPM^34^, Chinese Whispers^35^, DER^36^, Eigenvector^37^, EM^38^, Genetics Algorithm^16^, Girvan Newman^14^, Greedy Modularity^15^, Kcut^39^, Label Propagation^40^, Leiden^13^, Louvain^12^, Markov Clustering^41^, RBER Pots, RB Pots^42^[33], Significance^43^, Spinglass^42^, Surprise^44^, Walktrap^45^, Head tail^46^, LSWL+^47^, Paris, dan Regularized spectral^48^ using the library CDLIB^49,50^ dan Networkx^51^.

## Results and discussion

### Results on real-world datasets

**Figure 3:**
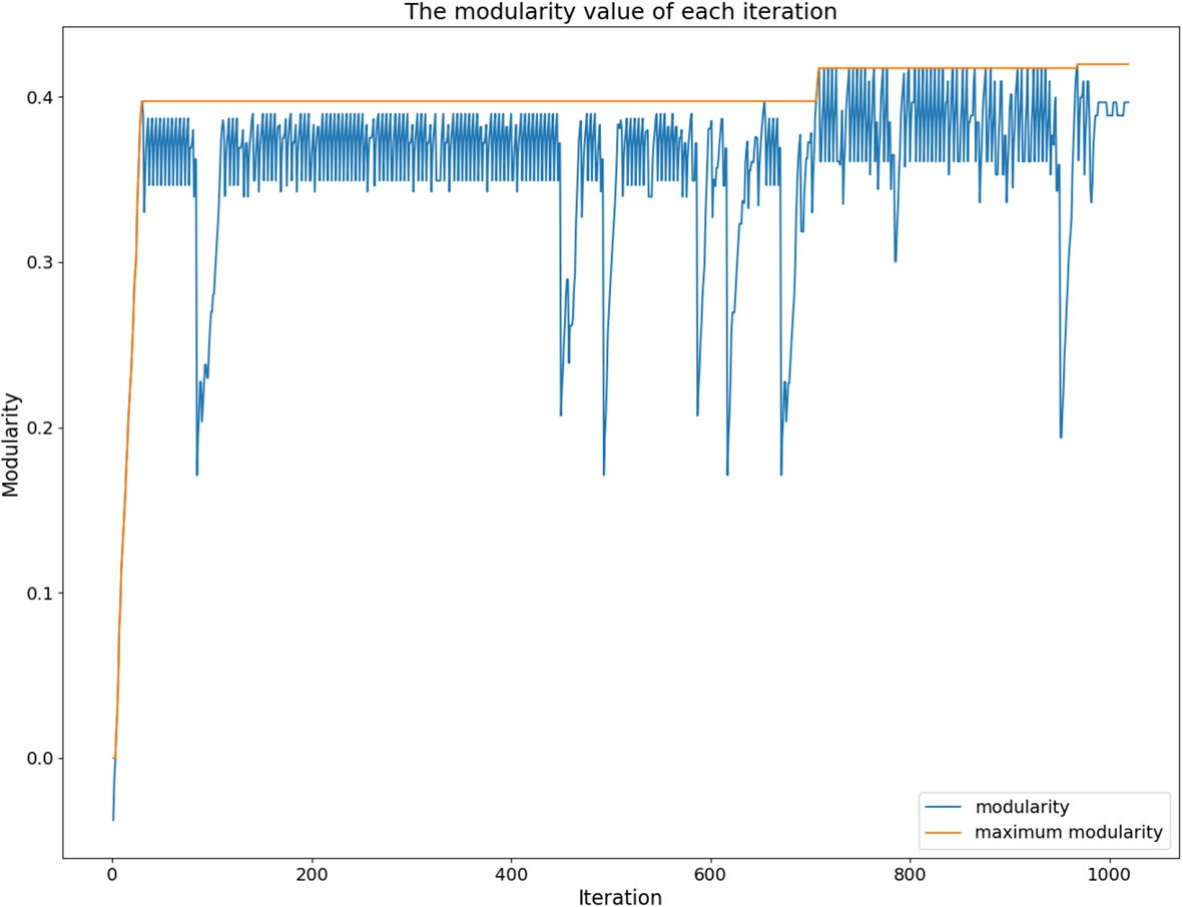
The value of the modularity of the DGM algorithm on the Zachary dataset (n=34), c=30.

Disassembly Greedy Modularity (DGM) is an extension of the Greedy Modularity algorithm with the addition of an exploration strategy. If the maximum number of iterations in Greedy Modularity is $n-1$, then in DGM, it is expressed as *pn* iterations. The higher the value of p, the greater the potential to obtain a community with better modularity; however, this causes the computation time to increase linearly for p. In each iteration, the possibility of greedy exploitation and exploration of the node community disassembly may occur, which is expressed as a percentage. In Fig.  3, the higher the number of iterations, the higher the potential to increase modularity, which is indicated by the yellow line. The blue line indicates the modularity value for each iteration. Modularity decreases when exploration occurs but increases again when exploitation occurs. The modularity used was the maximum modularity achieved across all the iterations.

Based on the exploratory strategy, all strategies in the DGM have increased modularity compared with the Greedy Modularity algorithm. The disassembly node and community strategy had a probability of 5 per cent in each iteration. A combination of five community and four node disassembly strategies resulted in 20 strategies. (Table 1). Compared with the Greedy Modularity algorithm, all combinations of strategies in the DGM can increase modularity. The best-combined strategy provides the greatest increase in the modularity. These combinations disassemble weak communities with random or low embeddedness nodes, disassemble low triangle partition ratio communities with non-triad nodes, and disassemble low internal edge density communities with low embeddedness nodes.

**Table 1:** Modularity in each combination of strategies in Zachary’s dataset.

Disassembly community strategy	Disassembly node strategy	Modularity
Random commmunity	Random node	0.4188
Random community	Weak node	0.4188
Random community	Non triad node	0.4020
Random community	Low embeddedness node	0.4172
Weak community	Random node	0.4198
Weak community	Weak node	0.4062
Weak community	Non triad node	0.3974
Weak community	Low embeddedness node	0.4198
High conductance community	Random node	0.4174
High conductance community	Weak node	0.3974
High conductance community	Non triad node	0.3974
High conductance community	Low embeddedness node	0.3974
Low triangle participation ratio community	Random node	0.4172
Low triangle participation ratio community	Weak node	0.3974
Low triangle participation ratio community	Non triad node	0.4198
Low triangle participation ratio community	Low embeddedness node	0.4020
Low internal edge density community	Random node	0.4110
Low internal edge density community	Weak node	0.4188
Low internal edge density community	Non triad node	0.4062
Low internal edge density community	Low embeddedness node	0.4198

**Figure 4:**
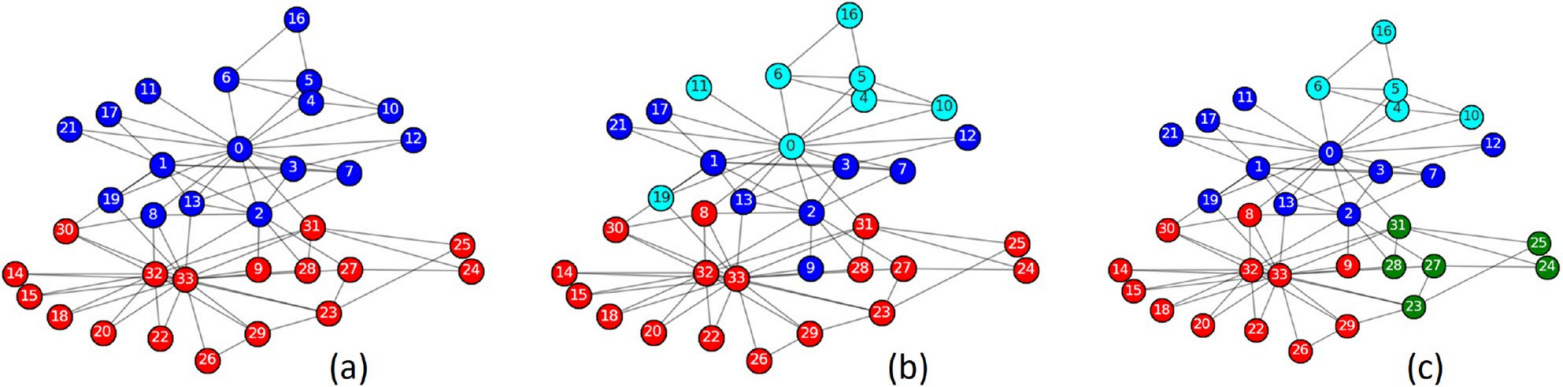
Visualization of community detection on (**a**) Zachary faction ground truth (**b**) Greedy Modularity (**c**) DGM.

In Zachary’s karate club, there are two groups: the group that follows the president and the group that follows the instructor, which is considered the reference or ground truth. However, based on the network topology structure, the community detection algorithm can identify more than two communities. Greedy Modularity can form three communities, whereas DGM can form four communities. The Zachary Karate Club obtained a modularity score of 0.3582, which was lower than that of the Greedy Modularity (0.3886) and DGM (0.4198). Greedy Modularity and DGM can reveal hidden communities or sub-communities that are not visible in factional groups. In the Greedy Modularity and DGM results, the blue and cyan communities are sub-communities against the blue communities in the ground truth. The red and green communities in the DGM were sub-communities relative to the red communities in the ground truth. At the node level, node eight is outside the red community in the ground truth and inside the red community in the Greedy Modularity and DGM. Node nine is inside the red community on the ground truth and outside the red community on the Greedy Modularity (Fig. 4).

The internal community density, which describes the density of a community in a network, has also increased. Density can be expressed as the number of edges in a community divided by the maximum number of edges that can occur in it. In the DGM, the density increased to 0.4508, whereas in the ground truth and greedy methods, it was 0.2463 and 0.3466, respectively, and the scaled density increased. However, an increase in modularity does not necessarily mean that other internal evaluations also increase because the number of communities increases, causing the average number of nodes in the community to decrease.

We also used several other datasets for the experiment, all of which experienced an increase in modularity compared with the Greedy Modularity algorithm. The highest increase in modularity is presented in Table 2.

We compared the performance of this DGM algorithm with those of 25 other algorithms, and the results are listed in Table 3 . It can be observed that the algorithm did not excel in all datasets. With the addition of exploration to Greedy Modularity, there was a significant improvement in almost all real-world datasets. Four of them were ranked first, namely Karate, Dolphins, Les Miserables, and Polbooks, which were ranked 10th, 8th, 6th, and 11th, respectively, in the Greedy Modularity algorithm. The other two, Adjnoun and Football, were ranked 4th and 8th, respectively, which were previously ranked 6th and 12th in the Greedy Modularity algorithm. This also occurs in information retrieval, hardware architect, and database datasets. This shows that both community and node disassembly significantly contribute to improving the modularity in community detection. DGM is one of the most stable algorithms in the top rankings, along with Spinglass, Leiden, Louvain, and RB Pots.

**Table 2:** The best combination of strategies across multiple reference data sets.

Dataset	Nodes	Disassembly community strategy	Disassembly node strategy	Modularity
Karate	34	Weak community	Random node	0.4198
Dolphins	62	Random community	Random node	0.5258
Lesmis	77	Random community	Weak node	0.5600
Polbooks	105	Random community	Weak node	0.5269
Adjnoun	112	Random community	Random node	0.3038
Football	115	High conductance community	Weak node	0.6006
Information retrieval (IR)	418	Weak community	Random node	0.6375
Hardware & Architecture (HA)	626	High conductance community	Random node	0.8309
Database (DB)	1006	High conductance community	Non triad node	0.7196

**Table 3:** Modularity comparison and ranking with 26 community detection algorithms for multiple datasets.

Method	${{\hbox {Modularity}}_{\textrm{rank}}}$								
	Karate	Dolphins	Les Miserable	Polbooks	Adjnoun	Football	Inf. Retrieval	H/W Architect.	Database
Spinglass	$0.420_{1}$	$0.525_{2}$	$0.559_{4}$	$0.525_{5}$	$0.309_{1}$	$0.603_{5}$	$0.648_{1}$	$0.836_{3}$	$0.753_{2}$
DGM	$0.420_{1}$	$0.526_{1}$	$0.560_{1}$	$0.527_{1}$	$0.304_{4}$	$0.601_{8}$	$0.638_{5}$	$0.831_{6}$	$0.720_{6}$
Leiden	$0.419_{3}$	$0.524_{3}$	$0.560_{1}$	$0.527_{1}$	$0.308_{2}$	$0.605_{1}$	$0.647_{2}$	$0.841_{2}$	$0.755_{1}$
Louvain	$0.419_{3}$	$0.524_{3}$	$0.558_{5}$	$0.527_{4}$	$0.300_{5}$	$0.605_{1}$	$0.642_{4}$	$0.834_{5}$	$0.747_{4}$
RB Pots	$0.419_{3}$	$0.524_{3}$	$0.560_{1}$	$0.527_{1}$	$0.308_{2}$	$0.605_{1}$	$0.646_{3}$	$0.841_{1}$	$0.753_{3}$
RBER Pots	$0.406_{6}$	$0.486_{12}$	$0.381_{18}$	$0.516_{8}$	$0.124_{16}$	$0.603_{4}$	$0.636_{6}$	$0.836_{4}$	$0.743_{5}$
Girvan Newman	$0.401_{7}$	$0.520_{6}$	$0.416_{16}$	$0.517_{7}$	$0.009_{20}$	$0.550_{15}$	$0.137_{24}$	$0.708_{14}$	$0.17_{22}$
Eigenvector	$0.393_{8}$	$0.491_{10}$	$0.532_{7}$	$0.467_{17}$	$0.243_{8}$	$0.493_{18}$	$0.559_{10}$	$0.797_{9}$	N/A
Genetics Algorithm	$0.393_{9}$	$0.437_{16}$	$0.506_{11}$	$0.424_{21}$	$0.186_{13}$	$0.442 _{20}$	$0.44_{17}$	$0.546_{18}$	$0.465_{16}$
Greedy Modularity	$0.389_{10}$	$0.506_{8}$	$0.556_{6}$	$0.495_{11}$	$0.277_{6}$	$0.580_{12}$	$0.630_{7}$	$0.824_{7}$	$0.714_{7}$
Surprise	$0.385_{11}$	$0.456_{13}$	$0.481_{12}$	$0.472_{14}$	$0.189_{12}$	$0.601_{9}$	$0.540_{11}$	$0.771_{12}$	$0.652_{11}$
Chinese Whispers	$0.372_{12}$	$0.456_{14}$	$0.479_{13}$	$0.487_{12}$	$0.000_{22}$	$0.602 _{7}$	$0.491_{13}$	$0.796_{10}$	$0.709_{9}$
Paris	$0.372_{12}$	$0.380_{20}$	$0.314_{21}$	$0.437_{20}$	$0.115_{17}$	$0.510_{17}$	$0.241_{22}$	$0.435_{22}$	$0.072_{23}$
Belief	$0.372_{14}$	$0.395_{18}$	$0.377_{19}$	$0.521_{6}$	$-0.070_{26}$	$0.573_{13}$	$0.625_{8}$	$0.789_{11}$	$0.7_{10}$
Regularized spectral	$0.372 _{14}$	$0.365 _{22}$	$0.232_{22}$	$0.444_{19}$	$0.192_{10}$	$0.375_{22}$	$0.235_{23}$	$0.26_{24}$	$0.279_{19}$
DER	$0.360_{16}$	$0.385_{19}$	$0.321_{20}$	$0.455_{18}$	$0.192_{11}$	$0.400_{21}$	$0.39_{18}$	$0.468_{20}$	$0.414_{18}$
Markov Clustering	$0.360 _{16}$	$0.455 _{15}$	$0.415 _{17}$	$0.514 _{9}$	$0.086_{19}$	$0.601_{9}$	$0.441_{16}$	$0.672_{16}$	$0.544_{15}$
Walktrap	$0.353_{18}$	$0.489_{11}$	$0.521_{10}$	$0.507_{10}$	$0.216_{9}$	$0.603_{6}$	$0.601_{9}$	$0.818_{8}$	$0.713_{8}$
Fluid	$0.343 _{19}$	$0.508 _{7}$	$0.524 _{9}$	$0.471 _{15}$	$0.260 _{7}$	$0.511 _{16}$	$0.54_{12}$	$0.512_{19}$	$0.565_{14}$
Label Propagation	$0.325 _{20}$	$0.499_{9}$	$0.527 _{8}$	$0.481 _{13}$	$0.000 _{22}$	$0.583 _{11}$	$0.482_{15}$	$0.73_{13}$	$0.649_{12}$
Head Tail	$0.264 _{21}$	$0.262 _{24}$	$0.150 _{24}$	$0.168 _{23}$	$0.181_{14}$	$0.187 _{23}$	$0.27_{20}$	$0.281_{23}$	$0.233_{21}$
Significance	$0.191 _{22}$	$0.355 _{23}$	$0.462 _{15}$	$0.391 _{22}$	$0.181 _{15}$	$0.568 _{14}$	$0.49_{14}$	$0.673_{15}$	$0.598_{13}$
EM	$0.176 _{23}$	$0.410 _{17}$	$0.466 _{14}$	$0.470 _{16}$	$-0.046 _{25}$	$0.473 _{19}$	$0.351_{19}$	$0.592_{17}$	$0.436_{17}$
AGDL	$0.079 _{24}$	$0.375 _{21}$	$0.152 _{23}$	$0.044 _{24}$	$0.098 _{18}$	$0.167 _{24}$	$0.268_{21}$	$0.463_{21}$	$0.251_{20}$
Kcut	$0.006 _{25}$	$0.011 _{25}$	$0.021 _{25}$	$0.014 _{25}$	$0.005 _{21}$	$0.005 _{25}$	$0.000_{25}$	$0.001_{25}$	$0.000_{25}$
CPM	-$0.050 _{26}$	$-0.021 _{26}$	$-0.024 _{26}$	$-0.014 _{26}$	$-0.016 _{24}$	$-0.009 _{26}$	-$0.004_{26}$	-$0.003_{26}$	-$0.002_{24}$

To address the above concerns related to the dataset size and diversity, we followed a holistic approach that encompasses multiple datasets displaying several characteristics to represent networks. It includes both scale-free networks, which are characterized by their degree distributions that have heavy tails, and random networks, which have a more uniform distribution of connections. Our explicit purpose in selecting these varied network types was to offer a broad representation of the possible variations in network characteristics. The results of our study revealed a consistent increase in modularity for these types of networks, providing additional support for the effectiveness and robustness of our community detection algorithm. Moreover, the fact that this performance is consistent across various network models implies the general relevance of our findings beyond the constraints set by the network size. This ensures that our results reflect a vast array of real-world network structures and behaviors embedded in the diversity of network types included in our analysis. This methodological approach highlights our commitment towards a comprehensive and inclusive research design that seeks to staff free not only the robustness of its findings but also their generalizability across different kinds of network structures.

**Figure 5:**
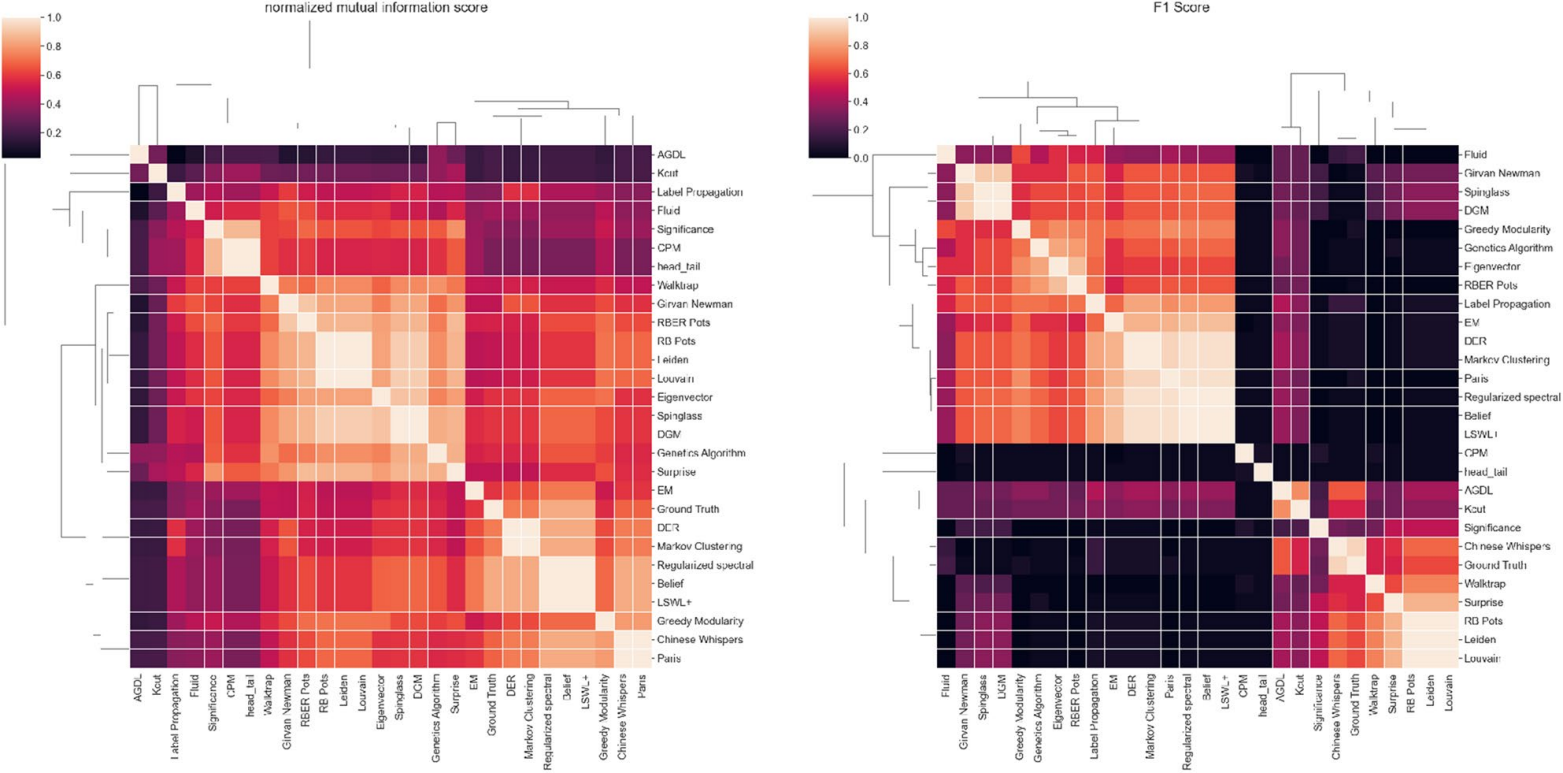
NMI and F1 Score visualization with heatmap.

A comparison of the results of community detection using NMI can be seen. In the Karate dataset, community generated by the DGM algorithm was the same as that generated by the Spinglass algorithm. Closer to both in a row are the Eigenvectors, Leiden, Louvain, and RB Pots, which have an NMI above 0.9. Figure 5 shows the NMI visualization of the community detection algorithms.

In generally, we computed the NMI for different datasets (Table 4). Although the similarity of the results between datasets and algorithms varies, the DGM is sufficiently stable to have similarities with Spinglass, as shown by the NMI value > 0.8 for each dataset. The DGM results also differ from those of the original algorithm, Greedy Modularity, with a similarity level varying between 0.5550 and 0.8435.

In our analysis, we used the F1 Score combined with Normalized Mutual Information (NMI) to compare the communities formed by various algorithms, and then another comparison against metadata is often used as a proxy for the ground truth (Fig. 5). In accordance with the NMI results, our Disassembly Greedy Modularity (DGM) algorithm shows closer co-linearity with the SpinGlass and Girvan–Newman algorithms but less correlation with the metadata-based ground truth. Therefore, one of the reasons for such a difference in classification has been rendered possible owing to the inherent ambiguity that lies in the relationship between metadata and the actual structural ground truth of networks. We identified two primary reasons for the failure to find a division that correlates well with metadata: (i) the relevance of metadata to network structure and (ii) the detected communities and metadata capturing potentially different aspects structure of the network^52^. Moreover, most real-life networks are generally complex, meaning that their nodes have multisource metadata, further creating havoc in data evaluation. It compels a misinformed perspective that there is a single community detection algorithm existing on the market that performs the best over others in all possible partitions, as advertised in^53^.

Most of the disassembly strategies employed in this study are related to the degree of a node; generally, higher internal degrees of a node have a greater influence on the community. In other cases, such nodes may be vital to a community and as a result, they are called community centers^54,55^. In other cases, it may mean placing the node on the periphery or outside the community owing to its higher external degree. This understanding has resulted in the development of strategies that may differentiate between internal and external degrees of nodes. By differentiating these degrees, our approach is capable of enabling nuanced identification and analysis of communities within networks.

**Table 4:** NMI between DGM and other methods, for multiple datasets.

Methods	Karate	Dolphins	Les Mis	Polbooks	Adjnoun	Football	Inf. Retrieval	H/W Architect.	Database
AGDL	0.1567	0.4858	0.2270	0.1068	0.0952	0.1357	0.2468	0.5193	0.285
Fluid	0.5175	0.8045	0.7806	0.5565	0.2715	0.5461	0.4092	0.5561	0.3113
Belief	0.6873	0.5344	0.4084	0.8068	0.1426	0.7274	0.7559	0.7328	0.4961
CPM	0.5449	0.5197	0.5792	0.4114	0.6233	0.5679	0.5116	0.5166	0.4901
Chinese Whispers	0.5878	0.5873	0.681	0.7396	0	0.9173	0.5874	0.7527	0.6673
DER	0.5856	0.5501	0.4007	0.6258	0.1797	0.5119	0.2564	0.3098	0.2096
Eigenvector	0.8965	0.7573	0.8084	0.645	0.3668	0.7208	0.5639	0.8021	N/A
EM	0.5655	0.6187	0.5511	0.6641	0.0999	0.6149	0.1842	0.3971	0.2171
Genetics Algorithm	0.8437	0.6764	0.7392	0.6721	0.4022	0.669	0.5627	0.6722	0.5551
Girvan Newman	0.8321	0.7628	0.7691	0.8489	0.0888	0.8598	0.3189	0.7235	0.332
Greedy Modularity	0.6341	0.8435	0.7962	0.8235	0.555	0.8029	0.6827	0.9144	0.7694
Kcut	0.3034	0.1623	0.1517	0.1016	0.0636	0.1058	0.0129	0.0215	0.0018
Label Propagation	0.5422	0.7669	0.6914	0.7096	0	0.8731	0.5637	0.7123	0.6402
Leiden	0.9233	0.7978	1	1	0.6465	0.9043	0.7529	0.913	0.7272
Louvain	0.9233	0.7978	0.9301	0.8513	0.553	0.9043	0.7089	0.8778	0.7423
Markov Clustering	0.5856	0.7822	0.6327	0.811	0.3141	0.8585	0.6083	0.6943	0.6178
RBER Pots	0.865	0.6866	0.6874	0.8025	0.4427	0.9078	0.6854	0.8764	0.7213
RB Pots	0.9233	0.7978	1	1	0.6465	0.9043	0.669	0.8994	0.7133
Significance	0.6519	0.5893	0.7184	0.5808	0.5865	0.8349	0.6014	0.6592	0.6042
Spinglass	1	0.9599	0.9532	0.8345	0.8233	0.8866	0.7573	0.8458	0.7172
Surprise	0.8592	0.6393	0.7019	0.6654	0.5463	0.8585	0.6183	0.7254	0.6249
Walktrap	0.762	0.7495	0.7283	0.8512	0.4974	0.8873	0.6609	0.8098	0.665
Head tail	0.5449	0.5197	0.5792	0.4114	0.6233	0.5911	0.5116	0.5166	0.4905
Paris	0.5878	0.5644	0.6766	0.6148	0.5944	0.751	0.3108	0.4682	0.1558
Regularized spectral	0.6873	0.4919	0.4406	0.5815	0.1603	0.458	0.2608	0.179	0.2285

### Results on synthetic datasets

**Figure 6:**
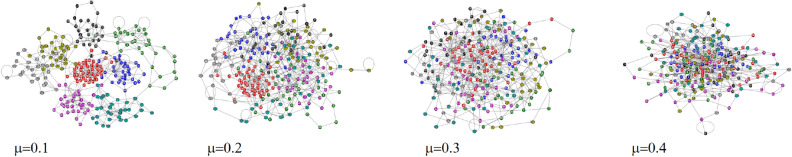
Visualisasi.

Synthetic dataset, using LFR, node degrees, and community size distributed by power law, with different exponential values for community degree and size $\gamma$ and $\beta$, respectively. The most important parameter for community detection was $\mu$. This parameter controls the number of edges between communities. If $\mu$ = 0, all links go to the nodes in the same community. Otherwise, if $\mu$ = 1 all links go to nodes in different communities. $\mu$ is above 0.5 external links between communities are larger than internal links. In this study we attempted to vary $\mu$ between 0.1 and 0.4, with 250 nodes, $\gamma$ = 3, and $\beta$ = 1.5 (Fig. 6).

**Table 5:** Comparison of modularity on synthetic datasets with $\mu$ variations with different community detection algorithms.

Method	Modularity (rank)			
	$\mu$ = 0.1	$\mu$ = 0.2	$\mu$ = 0.3	$\mu$ = 0.4
Leiden	$0.7974_{1}$	$0.6679_{1}$	$0.5346_{1}$	$0.5081_{2}$
RB Pots	$0.7974_{1}$	$0.6679_{1}$	$0.5341_{4}$	$0.5081_{2}$
DGM	$0.7974_{1}$	$0.6672_{3}$	$0.5342_{3}$	$0.5106_{1}$
Greedy Modularity	$0.7974_{1}$	$0.6539_{6}$	$0.5163_{6}$	$0.5008_{4}$
Louvain	$0.7972_{5}$	$0.6638_{5}$	$0.5234_{5}$	$0.4863_{6}$
RBER Pots	$0.7957_{6}$	$0.6668_{4}$	$0.5342_{2}$	$0.5005_{5}$
Walktrap	$0.7954_{7}$	$0.6114_{8}$	$0.4630_{8}$	$0.4149_{9}$
Belief	$0.7745_{8}$	$0.6487_{7}$	$0.1271_{15}$	-$0.0020_{18}$
Chinese Whispers	$0.7620_{9}$	$0.5548_{11}$	$0.0038_{18}$	$0.0000_{17}$
Eigenvector	$0.7387_{10}$	$0.5518_{12}$	$0.4501_{9}$	$0.4094_{10}$
Girvan Newman	$0.7119_{11}$	$0.5946_{9}$	$0.2264_{13}$	$0.1575_{14}$
GEMSEC	$0.6939_{12}$	$0.4837_{14}$	$0.4705_{7}$	$0.4689_{7}$
Label Propagation	$0.6405_{13}$	$0.5197_{13}$	$0.3886_{10}$	$0.0747_{15}$
Significance	$0.5705_{14}$	$0.4403_{15}$	$0.3416_{11}$	$0.3135_{13}$
DER	$0.4412_{15}$	$0.3518_{16}$	$0.2985_{12}$	$0.3166_{12}$
EM	$0.4085_{16}$	$0.2975_{17}$	$0.1721_{14}$	$-0.0212_{19}$
Surprise	$0.0302_{17}$	$0.0330_{18}$	$0.0181_{16}$	$0.3944_{11}$
CPM	$0.0283_{18}$	$0.0312_{19}$	$0.0163_{17}$	$0.0045_{16}$
Paris	$0.0000_{19}$	$0.5841_{10}$	$0.0000_{19}$	$0.4325_{8}$

On synthetic data sets, the DGM algorithm also significantly increases the modularity value compared to the Greedy Modularity algorithm (Table 5) . Within different variations of $\mu$, it experienced an increase in rank from 1-6 to 1-3.

### Computational complexity

One iteration for the greedy algorithm requires $O(n+m)$, and similarly for the community and node disassembly algorithms. However this algorithm required *cn* iterations. Therefore, the overall time complexity of this algorithm was $O(m+n)(n)$. Because the number of edges in the graph is greater than the number of nodes, the maximum complexity is $O(m^n)$.

## Conclusions and future works

The research shows that the DGM community detection algorithm can provide better results than the greedy algorithm, as calculated from its modularity, compared with some existing community detection algorithms.

The complexity of the algorithm is still quadratic with the number of vertices and linear with the number of iterations. It remains a challenge for this algorithm to solve big data. However, research is still open to improving the complexity such that it can be made close to linear by changing the data structure. Furthermore, better strategies can be developed, both for node and community disassembly, such that fewer iterations are required to produce better modularity. This algorithm can be used to solve real problems, such as community detection in the field of bioinformatics, namely clustering essential proteins in cancer and examining functional associations with the resulting communities.

## Data Availability

Publicly available datasets were analyzed in this study. The real world dataset can be accessed from http://www-personal.umich.edu/~mejn/netdata/ and https://github.com/vlivashkin/community-graphs. LFR synthetic dataset generated via the networkx library available at https://networkx.org/.

## References

[1] Barabási A-L (2016). Network Science.

[2] Shang KK, Small M, Wang Y, Yin D, Li S (2020). A novel metric for community detection. Europhys. Lett..

[3] Chakraborty T, Dalmia A, Mukherjee A, Ganguly N (2017). Metrics for community analysis: A survey. ACM Comput. Surv..

[4] Chen M, Kuzmin K, Szymanski BK (2014). Community detection via maximization of modularity and its variants. IEEE Trans. Comput. Soc. Syst..

[5] Zhu J (2020). A no self-edge stochastic block model and a heuristic algorithm for balanced anti-community detection in networks. Inf. Sci..

[6] Huang M, Jiang Q, Qu Q, Rasool A (2021). An overlapping community detection approach in ego-splitting networks using symmetric nonnegative matrix factorization. Symmetry.

[7] Gutiérrez, I., Gómez, D., Castro, J. & Espínola, R. A new community detection problem based on bipolar fuzzy measures. 10.1007/978-3-030-88817-6_11 (2022).

[8] Tabarzad MA, Hamzeh A (2017). A heuristic local community detection method (HLCD). Appl. Intell..

[9] Yin Y, Zhao Y, Li H, Dong X (2021). Multi-objective evolutionary clustering for large-scale dynamic community detection. Inf. Sci..

[10] Zhang X, Wang C, Su Y, Pan L, Zhang HF (2017). A fast overlapping community detection algorithm based on weak cliques for large-scale networks. IEEE Trans. Comput. Soc. Syst..

[11] Zhou X, Yang K, Xie Y, Yang C, Huang T (2019). A novel modularity-based discrete state transition algorithm for community detection in networks. Neurocomputing.

[12] Blondel VD, Guillaume J-L, Lambiotte R, Lefebvre E (2008). Fast unfolding of communities in large networks. J. Stat. Mech. Theory Exp..

[13] Traag VA, Waltman L, van Eck NJ (2019). From Louvain to Leiden: Guaranteeing well-connected communities. Sci. Rep..

[14] Girvan M, Newman ME (2002). Community structure in social and biological networks. Proc. Natl. Acad. Sci. U. S. A..

[15] Newman ME (2004). Fast algorithm for detecting community structure in networks. Phys. Rev. E Stat. Phys. Plasmas Fluids Relat. Interdiscip. Top..

[16] Ghoshal, A. K., Das, N., Bhattacharjee, S. & Chakraborty, G. A fast parallel genetic algorithm based approach for community detection in large networks. 10.1109/COMSNETS.2019.8711127 (2019).

[17] Zeng X, Wang W, Chen C, Yen GG (2020). A consensus community-based particle swarm optimization for dynamic community detection. IEEE Trans. Cybern..

[18] Rustamaji HC (2022). A network analysis to identify lung cancer comorbid diseases. Appl. Netw. Sci..

[19] Fortunato S, Barthélemy M (2007). Resolution limit in community detection. Proc. Natl. Acad. Sci..

[20] Guo, J., Singh, P. & Bassler, K. E. Resolution limit revisited: Community detection using generalized modularity density (2020). arXiv:2012.14543.

[21] Miyauchi A, Kawase Y (2016). Z-score-based modularity for community detection in networks. PLoS One.

[22] Fortunato S, Hric D (2016). Community detection in networks: A user guide. Phys. Rep..

[23] He K, Li Y, Soundarajan S, Hopcroft J E (2018). Hidden community detection in social networks. Inf. Sci..

[24] Chattopadhyay S, Ganguly D (2021). Node2vec with weak supervision on community structures. Pattern Recognit. Lett..

[25] Orman, G. K., Labatut, V. & Cherifi, H. Qualitative comparison of community detection algorithms. In *Digital Information and Communication Technology and Its Applications: International Conference, DICTAP 2011, Dijon, France, June 21-23, 2011, Proceedings, Part II*. 10.1007/978-3-642-22027-2_23 (2011).

[26] Tandon A (2021). Community detection in networks using graph embeddings. Phys. Rev. E.

[27] Lancichinetti A, Fortunato S, Radicchi F (2008). Benchmark graphs for testing community detection algorithms. Phys. Rev. E Stat. Nonlinear Soft Matter Phys..

[28] Suyanto S, Ariyanto AA, Ariyanto AF (2022). Komodo mlipir algorithm. Appl. Soft Comput..

[29] Orman GK, Labatut V, Cherifi H (2012). Comparative evaluation of community detection algorithms: A topological approach. J. Stat. Mech. Theory Exp..

[30] Yang J, Leskovec J (2015). Defining and evaluating network communities based on ground-truth. Knowl. Inf. Syst..

[31] Zhang, W., Wang, X., Zhao, D. & Tang, X. Graph degree linkage: Agglomerative clustering on a directed graph. In *Lecture Notes in Computer Science (including subseries Lecture Notes in Artificial Intelligence and Lecture Notes in Bioinformatics)* 7572 LNCS, 428–441. 10.1007/978-3-642-33718-5_31 (2012).

[32] Parés, F. *et al.* Fluid communities: A competitive, scalable and diverse community detection algorithm. In *Complex Networks & Their Applications VI: Proceedings of Complex Networks 2017 (The Sixth International Conference on Complex Networks and Their Applications)* 229–240 (Springer International Publishing, 2018). 10.1007/978-3-319-72150-7_19.

[33] Zhang P, Moore C (2014). Scalable detection of statistically significant communities and hierarchies, using message passing for modularity. Proc. Natl. Acad. Sci. U. S. A..

[34] Traag VA, Van Dooren P, Nesterov Y (2011). Narrow scope for resolution-limit-free community detection. Phys. Rev. E Stat. Nonlinear Soft Matter Phys..

[35] Biemann, C. Chinese whispers—An efficient graph clustering algorithm and its application to natural language processing problems. In *Proceedings of TextGraphs: The 1st Workshop on Graph-Based Methods for Natural Language Processing* 73–80 (2020).

[36] Kozdoba M, Mannor S, Cortes C, Lawrence N, Lee D, Sugiyama M, Garnett R (2015). Community detection via measure space embedding. Advances in Neural Information Processing Systems.

[37] Newman ME (2006). Finding community structure in networks using the eigenvectors of matrices. Phys. Rev. E Stat. Nonlinear Soft Matter Phys..

[38] Chang Z, Yin X, Jia C, Wang X (2018). Mixture models with entropy regularization for community detection in networks. Phys. A Stat. Mech. Appl..

[39] Ruan, J. & Zhang, W. An efficient spectral algorithm for network community discovery and its applications to biological and social networks. In *Proceedings—IEEE International Conference on Data Mining, ICDM* 643–648, 10.1109/ICDM.2007.72 (2007).

[40] Cordasco, G. & Gargano, L. Community detection via semi-synchronous label propagation algorithms. In *2010 IEEE international workshop on: business applications of social network analysis (BASNA)* 1–8 (IEEE, 2010). 10.1109/BASNA.2010.5730298.

[41] Enright AJ, Van Dongen S, Ouzounis CA (2002). An efficient algorithm for large-scale detection of protein families. Nucleic Acids Res..

[42] Reichardt J, Bornholdt S (2006). Statistical mechanics of community detection. Phys. Rev. E Stat. Nonlinear Soft Matter Phys..

[43] Traag VA, Krings G, Van Dooren P (2013). Significant scales in community structure. Sci. Rep..

[44] Traag VA, Aldecoa R, Delvenne JC (2015). Detecting communities using asymptotical surprise. Phys. Rev. E Stat. Nonlinear Soft Matter Phys..

[45] Pons P, Latapy M (2006). Computing communities in large networks using random walks. J. Graph Algorithms Appl..

[46] Jiang B, Ma D (2015). Defining least community as a homogeneous group in complex networks. Phys. A Stat. Mech. Appl..

[47] Luo F, Wang JZ, Promislow E (2008). Exploring local community structures in large networks. Web Intell. Agent Syst..

[48] Zhang Y, Rohe K (2018). Understanding regularized spectral clustering via graph conductance. Adv. Neural Inf. Process. Syst..

[49] Rossetti G, Milli L, Cazabet R (2019). CDLIB: A python library to extract, compare and evaluate communities from complex networks. Appl. Netw. Sci..

[50] Rossetti, G. CDlib—Community Discovery Library—CDlib—Community Discovery library (2019).

[51] Hagberg, A. A., Schult, D. A. & Swart, P. J. Exploring network structure, dynamics, and function using NetworkX. In *7th Python in Science Conference (SciPy 2008)* 11–15 (2008).

[52] Peel L, Larremore DB, Clauset A (2017). The ground truth about metadata and community detection in networks. Sci. Adv..

[53] Chakraborty, T., Cui, Z. & Park, N. Metadata vs. ground-truth: A myth behind the evolution of community detection methods. In *Companion Proceedings of the The Web Conference 2018, WWW ’18* 45–46, 10.1145/3184558.3186921 (International World Wide Web Conferences Steering Committee, Republic and Canton of Geneva, CHE, 2018).

[54] Blondel VD, Guillaume J-L, Lambiotte R (2008). Local leaders in random networks. Phys. Rev..

[55] Shang, F. *et al.* Local dominance unveils clusters in networks. arXiv:2209.15497v1 (2022).

